# A New Signature of Sarcoma Based on the Tumor Microenvironment Benefits Prognostic Prediction

**DOI:** 10.3390/ijms24032961

**Published:** 2023-02-03

**Authors:** Guanran Zhang, Aiwen Jian, Yundi Zhang, Xiaoli Zhang

**Affiliations:** 1 Key Laboratory for Experimental Teratology of Ministry of Education, Department of Histology & Embryology, School of Basic Medical Sciences, Shandong University, Jinan 250012, China; 2 Key Laboratory of Molecular Oncology, Department of Medical Oncology, Chinese Academy of Medical Sciences & Peking Union Medical College, Beijing 100021, China

**Keywords:** tumor microenvironment, sarcoma, immune-related genes, immunotherapy, prognosis

## Abstract

Sarcomas are a group of malignant tumors derived from mesenchymal tissues that display complex and variable pathological types. The impact of the immune properties of the tumor microenvironment (TME) on the prognosis, treatment, and management of sarcomas has attracted attention, requiring the exploration of sensitive and accurate signatures. In this study, The Cancer Genome Atlas (TCGA) database was searched to screen for an RNA sequencing dataset, retrieving 263 sarcoma and 2 normal samples with survival data. Genes associated with immune regulation in sarcomas were retrieved from the Tumor Immune Estimation Resource database to estimate tumor purity and immune cell infiltration levels. The samples were then divided into the immune-high and immune-low groups. Then, we screened for differentially expressed genes (DEGs) between the two groups. The intersection between immune-related genes and DEGs was then determined. Univariate Cox and least absolute shrinkage and selection operator analyses were used to select ideal genes for prognostic prediction and subsequent construction of a risk signature. A survival analysis was performed to reveal the dissimilarity in survival between the high- and low-score groups. Finally, a nomogram was generated to verify the accuracy and reliability of the signature. Through Estimation of STromal and Immune cells in MAlignant Tumour tissues using Expression (ESTIMATE) analysis, high ESTIMATE, and low tumor purity were significantly associated with a favorable prognosis. Moreover, a total of 5259 DEGs were retrieved, the majority of which were downregulated. In total, 590 immune-associated genes overlapped with the DEGs, among which nine hub genes were identified. Finally, two candidate genes, *ACVR2B* and *NFYA*, were identified, based on which a risk signature was constructed. The risk signature constructed in this study is accurate and reliable for the prognostic prediction and phenotyping of sarcomas.

## 1. Introduction

Sarcomas represent a group of neoplasms with diverse genomic backgrounds, and are usually associated with high malignancy, severe conditions, and a poor prognosis. There are various subtype classification criteria for sarcomas. The most traditional histological classification categorizes over 70 subtypes of sarcomas into 2 main types according to the origin of tissue in its primary form. These include sarcomas in bones, sarcomas in soft tissues, and many pediatric sarcomas. Bone sarcomas include osteosarcoma and chondrosarcoma. Soft tissue sarcomas include liposarcoma, undifferentiated pleomorphic sarcoma, leiomyosarcoma, rhabdomyosarcoma, and fibrosarcoma [1,2]. Two distinct subtypes can be classified according to genetic criteria such as karyotypes and chromosomal changes. The first subtype of sarcomas has a normal karyotype and a discrete chromosomal structure and includes most childhood sarcomas. The second subtype is characterized by considerably complex karyotypes with prominent structural and quantitative alterations and occurs commonly in adults [3,4]. The symptoms that cause concern often depend on the origin of the sarcoma and how fast it grows. The prognosis is usually poor when sarcomas originate in relatively hidden areas, such as in the case of retroperitoneal sarcomas, as the tumors are often already large by the time the patient visits a doctor, and most of them are already in advanced stages. The wide variety, low incidence, and difficulty in evaluating and managing new cases of sarcomas often lead to delays in treatment. Sarcomas that metastasize or recur due to treatment delay are more difficult to treat and have a worse prognosis, with 5-year survival rates often lower than 20% [5,6]. In recent years, numerous studies have found an increase in the global incidence of sarcomas, calling for the development of comprehensive assessment tools to optimize treatment management [7].

The traditional treatment of sarcomas includes surgery, preoperative and postoperative radiotherapy, and chemotherapy. However, the prognosis is far from satisfactory, with no significant benefit in disease-free survival or overall survival observed [8,9]. In recent decades, immunotherapy has rapidly gained attention and become the most promising anticancer strategy. 

Immune checkpoint blockade (ICB) therapy promotes T-cell-mediated antitumor effects by using antibodies targeting immune checkpoint receptors or ligands to inhibit receptor-ligand interactions. Two types of immune checkpoints are the most accessible to ICB antibodies. Through clinical trials, ICB has been demonstrated to be effective in the treatment of different kinds of solid tumors [10,11,12]. The success of these clinical trials has greatly contributed to the study of ICB for sarcoma treatment, which has further promoted the investigation of the sarcoma tumor microenvironment (TME). It has been demonstrated that the immune properties of the TME are highly correlated with clinical manifestations, prognosis, and response to immunotherapy [13,14,15,16]. Gu et al. showed that immune-related genes have a high prognostic value and are suitable for constructing prognostic markers, and that their expression levels are closely related to tumorigenesis and TME characteristics [17]. Further understanding of the general immunological background and TME of sarcomas can assist in the development of immunotherapy.

In our study, a deconvolution algorithm was employed on the RNA sequencing data of sarcoma samples retrieved from an online database to evaluate the immune-related scores for each sample. Immune-associated genes in two separate groups, divided according to the single-sample gene set enrichment analysis (ssGSEA) analysis, were screened for further investigation. Based on the functions, prognostic value, and correlations between the expression levels of these genes, along with the immune infiltration level, a comprehensive immune signature was constructed. The signature was further tested for its capacity to predict sarcoma prognosis and response to immunotherapy. The results of this research might provide assistance in the improvement of overall survival and in the design of precise individualized therapy.

## 2. Results

### 2.1. Landscape of the Overall Immune Signature

**Figure 1:**
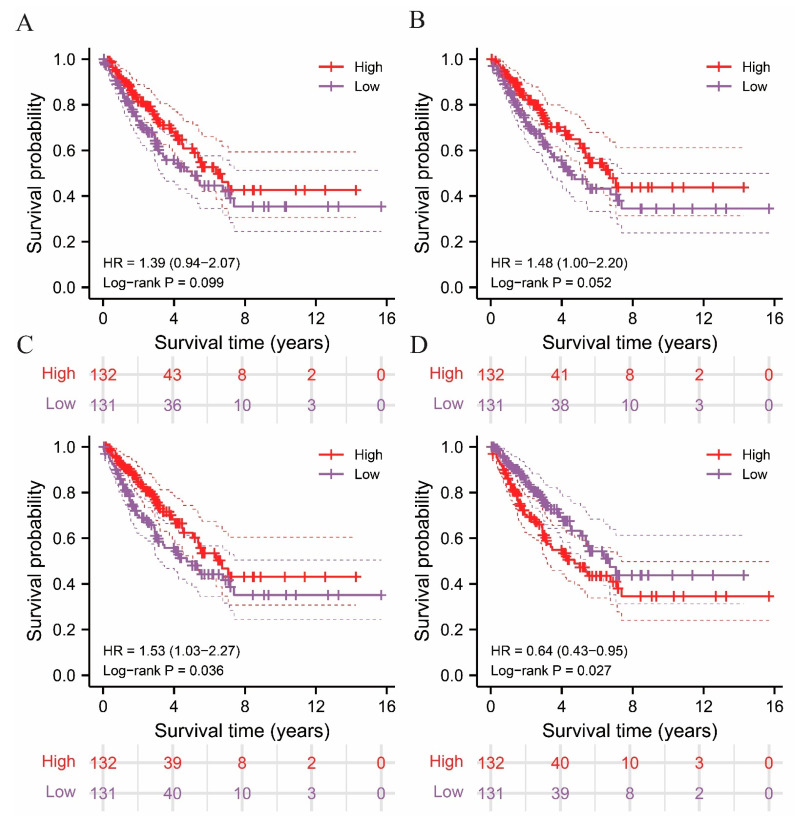
Survival analysis performed in high and low immune score groups using ESTIMATE algorithm and illustrated in Kaplan–Meier (K-M) curves. (**A**) Immune score, (**B**) stromal score, (**C**) ESTIMATE score, and (**D**) tumor purity score.

Based on the stromal and immune signatures of the sarcoma samples, estimation of stromal and immune cells in malignant tumor tissues using the expression data (ESTIMATE) algorithm was applied to reliably reveal the infiltration levels of tumor and non-tumor cells in sarcoma samples. The immune, stromal, ESTIMATE, and tumor purity scores of the sarcoma samples were calculate and analyzed (Table S3). Based on the above signatures, the 263 samples were separated into two groups on the basis of their average values, and were named the high-score and low-score groups, respectively. Subsequently, Kaplan-Meier analysis was conducted to explore the association between the immune, stromal, ESTIMATE, and tumor purity scores and the future status of the sarcoma patients (Figure 1A–D). A prominent difference in overall survival (OS) was observed between the upper and lower halves of the ESTIMATE and tumor purity scores, where high ESTIMATE (*p* = 0.036) and low tumor purity (*p* = 0.027) scores were significantly associated with a higher survival rate. However, this distinction was not observed in the survival curves based on immune (*p* = 0.099) and stromal (*p* = 0.052) scores. As demonstrated in Figure 1, the survival of patients with sarcoma is significantly associated with ESTIMATE and tumor purity score, where the survival rate of patients correlated positively with ESTIMATE score and negatively with tumor purity. The data acquired from the above analysis indicate a possibility of utilizing the immune-associated scores of sarcomas in prognostic prediction. Subsequent ssGSEA analysis was performed for further validation.

**Figure 2:**
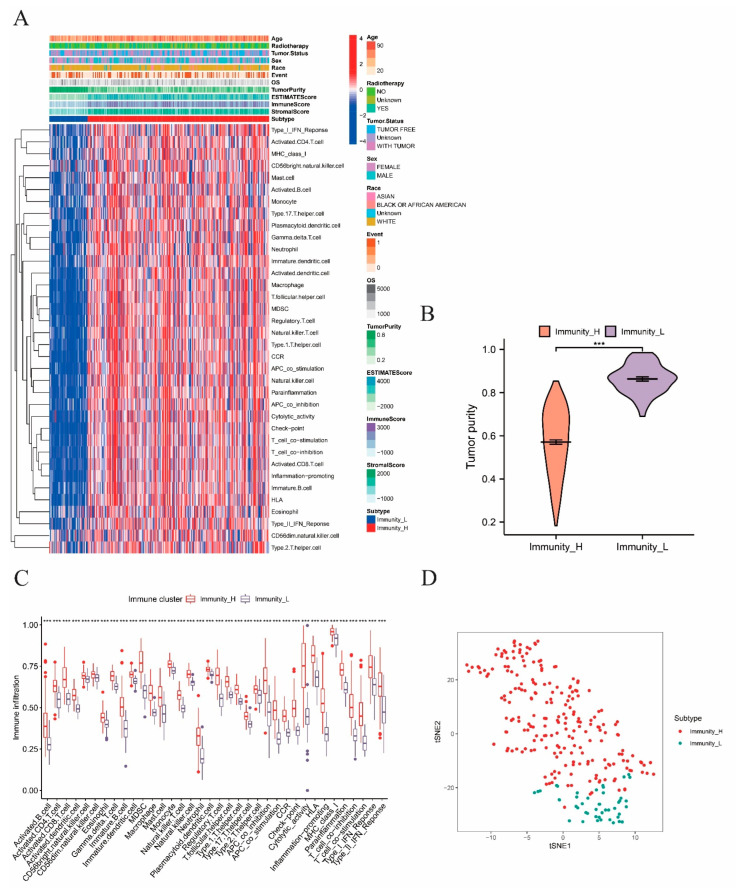
Identification of immune subtypes and differential analysis. The symbols *** represent *p*-values less than 0.001. (**A**) The ssGSEA analysis performed on the 263 samples to divide the samples into two groups according to 30 ssGSEA scores of each sample. (**B**) Differential analysis on tumor purity score between immunity−high and −low groups. (**C**) Box plot presenting the immune−cell infiltration levels of the immunity−high and −low groups, with the red boxes and blue boxes representing immunity−high and −low groups, respectively. (**D**) The t−SNE analysis on the two immune subtypes, with the red dots and blue dots representing immunity−high and −low groups, respectively.

Subsequently, ssGSEA was utilized to examine the impact that immune activation had on sarcoma progression and prognosis of patients with sarcoma. The immune signatures of 30 immune cell types and clinical features including age, radiotherapy, tumor status, sex, race, event, and OS are shown in Figure 2A. In line with the total score in individual, unsupervised clustering separated the samples into two distinct groups with obvious differences in immune signatures. The group with a significant abundance of various immune signatures, including a type I interferon (IFN) response, CD4 T and B cell activation, T follicular helper cells, and Treg enrichment, was named the immunity-high group. The other group with lower immune activation was therefore named the immunity-low group. To investigate the dissimilarities in TME, immune cell activation, and the level of immune and tumor cells infiltration between the upper and lower halves, several analyses were performed on the sarcoma TME. The immunity-low group had significantly higher tumor purity, as expected (Figure 2B). Hence, a correlation between high immune activation and low tumor purity was suggested. The immunity-high group also showed noticeably a higher expression of immune checkpoints and promotion of inflammation (Figure 2C). In the subsequent t-distributed stochastic neighbor embedding (t-SNE) analysis, two clusters with different immunity characteristics were clearly differentiated and visualized (Figure 2D), confirming the existence of immune differences among each sarcoma sample and the validity of classifying sarcoma patients according to different immune cell infiltration levels. Moreover, the two immune subtypes exhibited significantly distinct features, as demonstrated in Figure S1A. Therefore, we suspected that the immune subtypes discovered above could be completely distinguished from each other on the basis of both immune and genetic differences. This assumption was verified by principal component analysis (PCA) (Figure S1B).

### 2.2. Screening and Evaluation of Differentially Expressed Genes (DEGs)

**Figure 3:**
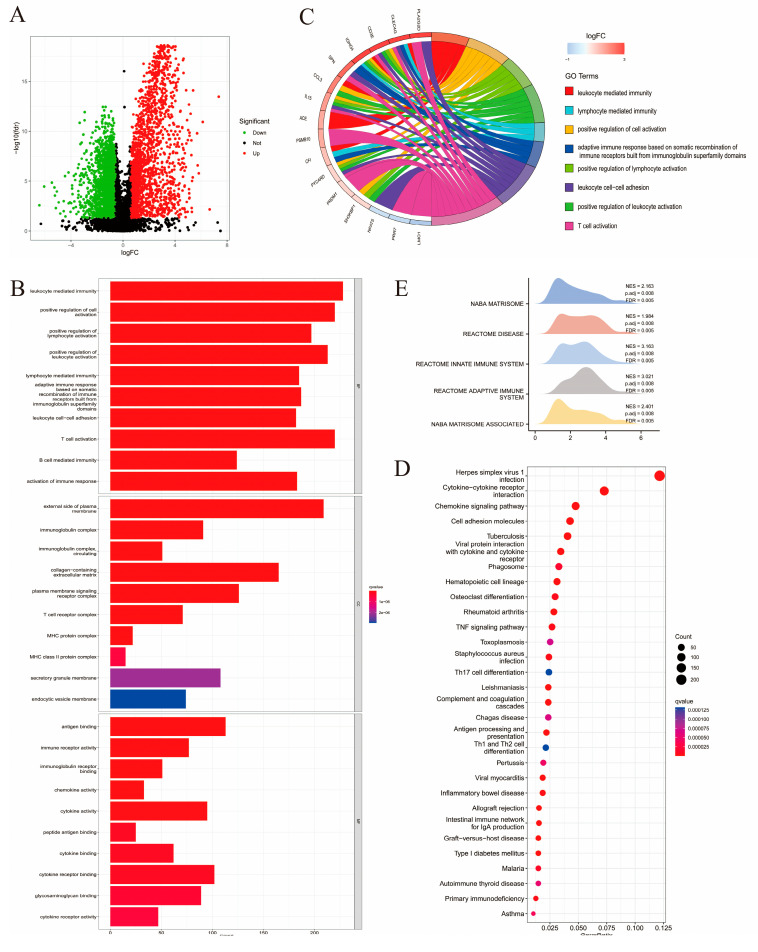
Enrichment analysis performed on DEGs obtained from the two immune subtypes. (**A**) Volcano plot demonstrating the regulation in DEGs expression, with the green, black, red dots representing down-, not-, and upregulation, respectively. (**B**) The histogram showing the top 10 enriched GO biological processes (BP), cell components (CC), and molecular functions (MF). (**C**) Circle graph presenting the top eight enriched GO terms. (**D**) Bubble chart demonstrating the top 30 enriched KEGG pathways, with the size of dot representing the enriched count. (**E**) Mountain plot listing the top five pathways with the most DEG enrichment according to Gene Set Enrichment Analysis (GSEA).

Genes that exhibited a different expression level between the two immune subtypes were screened using the differential expression analysis. A total of 5259 DEGs were screened for further analysis (Table S4), among which 3374 were downregulated (64.16%), while the remaining 1885 were upregulated (35.84%) (Figure 3A). Enrichment analysis was conducted on DEGs to obtain functional annotations. Through Gene Ontology (GO) annotation and Kyoto Encyclopedia of Genes and Genomes (KEGG) pathway enrichment analyses, the number of DEGs was found significantly enriched for 1052 biological processes (BPs), 600 types of cell components (CCs), 650 molecular functions (MFs), and 65 KEGG pathways.

As visualized using a bar chart, the top 10 enriched biological pathways were mostly immune-related including leukocyte-mediated immunity, T-cell activation, and leukocyte cell-cell adhesion (Figure 3B). In the GO circle graph, the top eight GO terms ranked by gene abundance are presented (Figure 3C). Enriched KEGG pathways included herpes simplex virus 1 infection, cytokine-cytokine receptor (CCR) interaction, chemokine signaling pathways, and cell adhesion molecules (Figure 3D). As indicated by the above enrichment analysis results, DEGs were found to be strongly associated with immune activation. GSEA was performed based on the DEGs to further investigate the differences in BP enrichment between the two groups with different immune features (Figure 3E). A total of 460 enriched pathways were obtained, the top five of which included the NABA matrisome (NES = 2.163, P.adj = 0.008, FDR = 0.005), reactome disease (NES = 1.984, P.adj = 0.008, FDR = 0.005), reactome innate immune system (NES = 3.163, P.adj = 0.008, FDR = 0.005), reactome adaptive immune system (NES = 3.021, P.adj = 0.008, FDR = 0.005), and NABA matrisome-associated pathways (NES = 2.401, P.adj = 0.008, FDR = 0.005). These results indicate that immune activation in the TME may play an important role in sarcomas.

### 2.3. Identical Gene Analyses for Immune-Related DEGs and Functional Annotations

**Figure 4:**
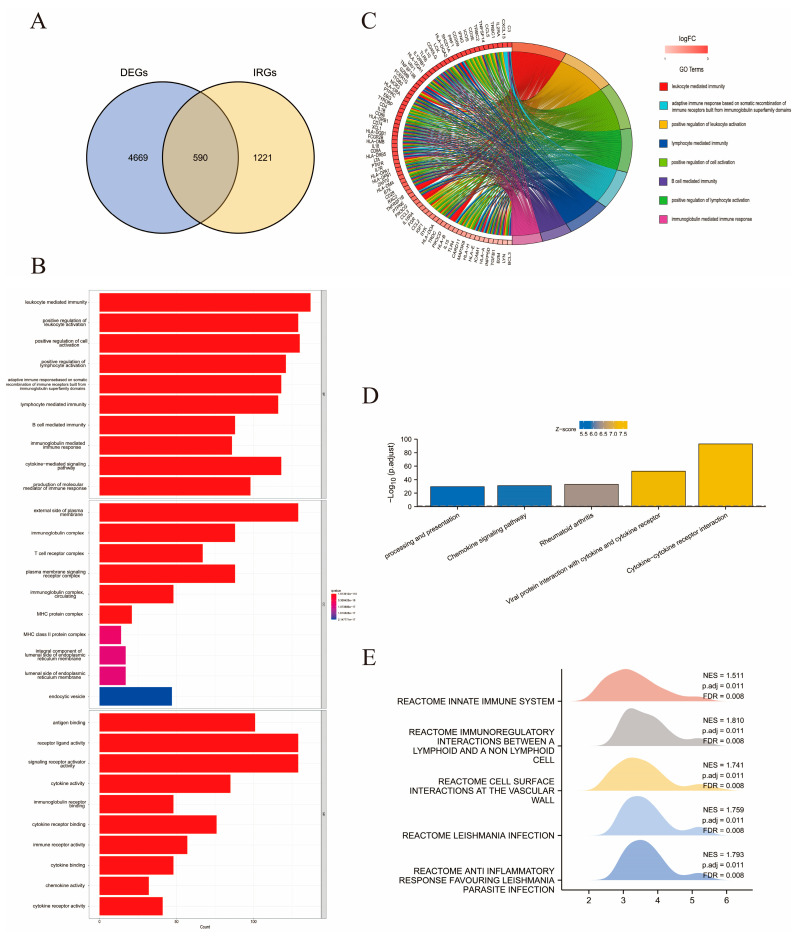
Identification of immune-related DEGs and enrichment analysis. (**A**) Venn gram showing the overlapping of 5259 DEGs and 1811 IRGs by 590 immune-related DEGs. (**B**) The histogram showing the top 10 enriched GO terms. (**C**) Circle graph presenting the top eight enriched GO terms. (**D**) Bar chart indicating the top five enriched KEGG pathways. (**E**) Mountain plot listing the top five pathways with the most gene enrichment according to GSEA analysis.

A total of 1811 immune-related genes (IRGs) were identified from the Tumor Immune Estimation Resource (TIMER) database (Table S1). The duplicate genes between DEGs and IRGs are shown in a Venn diagram (Figure 4A). As a result, 590 overlapping genes were identified and denoted as immune-related DEGs (Table S5). Enrichment analysis of the 590 genes was carried out to obtain functional annotations, including BPs and biological pathways. In total, 1693 GO terms were composed of 1517 BPs, 64 CCs, and 112 MFs. As visualized in the GO bar chart and Circos graph in Figure 4B,C, prominent enrichment categories of these immune-related DEGs were identified as leukocyte-mediated immunity, upregulation of leukocyte activation, and lymphocyte activation. Among the 101 enriched KEGG pathways identified, the top five enriched pathways were CCR interaction, viral protein interaction with CCR, rheumatoid arthritis, chemokine signaling pathway, and processing and presentation (Figure 4D). Subsequent GSEA identified the top five pathways with the most enriched genes, which from most to least were reactome immunoregulatory interactions between a lymphoid and a non-lymphoid cell (NES = 1.1810, P.adj = 0.011, FDR = 0.008), reactome anti-inflammatory response favoring *Leishmania* parasite infection (NES = 1.793, P.adj = 0.011, FDR = 0.008), reactome *Leishmania* infection (NES = 1.759, P.adj = 0.011, FDR = 0.008), reactome cell surface interactions at the vascular wall (NES = 1.741, P. adj = 0.011, FDR = 0.008), and reactome innate immune system (NES = 1.511, P.adj = 0.011, FDR = 0.008). Similar to the above results, immune activation, especially lymphoid and T cell activation, is important for sarcoma development (Figure 4E).

To investigate interactions among immune-related DEG-encoded proteins retrieved from the Search Tool for the Retrieval of Interacting Genes/Proteins (STRING) database, a protein-protein interaction (PPI) network was generated (Figure S1C). A total of 5259 nodes and 1411 interactions were included in the network, and the topological properties of the nodes are listed in Table S6. Subsequently, to further investigate the interactions and correlations among these immune-related DEGs, an interaction network was generated (Figure S1D). A significant association was found between multiple immune-related DEGs. The interactions between *IRF7*, *STAT1*, *ISG15*, *MX1*, *RSAD2*, and other immune-related DEGs were especially significant. 

The interaction network contained three interferon regulatory factors (IRFs), including *IRF1*, *IRF5*, and *IRF7*. These genes were among the hub nodes and displayed a significant influence over other genes. In addition, IFN-stimulated gene 15 (*ISG15*), myxovirus resistance 1 (*MX1*), proteasome (prosome, macropain) subunit beta type 8 (*PSMB8*), radical S-adenosyl methionine domain containing 2 (*RSAD2*), signal transducer and activator of transcription 1 (*STAT1*), and bone marrow stromal cell antigen 2 (*BST2*) were among the hub genes. The hub nodes were then ranked using the maximal clique centrality (MCC) method, the top ten of which are listed in Table S7 for further analysis. 

**Figure 5:**
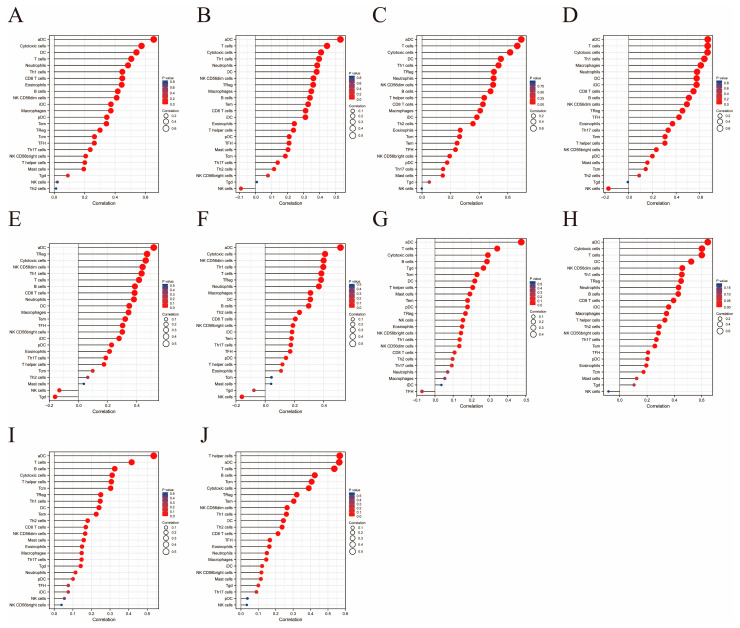
Immune cell infiltration analysis performed on the ten hub genes, presented in the form of lollipop charts. (**A**–**J**) Lollipop charts for BST2, IFITM1, IRF1, IRF5, IRF7, ISG15, MX1, PSMB8, RSAD2, STAT1, respectively.

**Figure 6:**
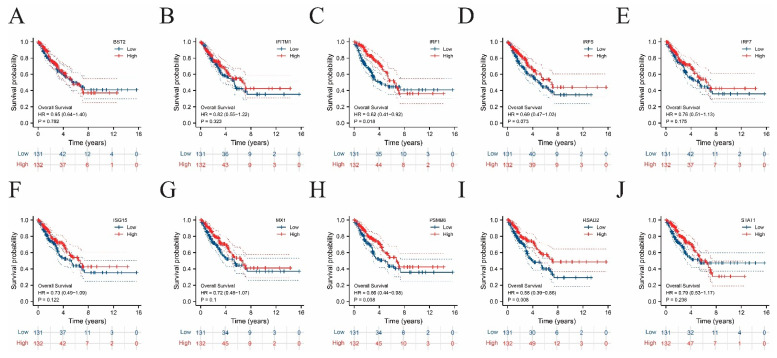
Survival analysis performed on the ten hub genes.

To explore the immune and clinical characteristics of the top 10 hub genes, the immune infiltration levels and OS of these genes were analyzed (Figure 5 and Figure 6 and Table S8). In all ten cases, the increases in dendritic and T-cell infiltration levels were the most significant, again underscoring the relationship between T-cell enrichment in TME and sarcoma development. Meanwhile, almost no correlation was detected between natural killer cells and hub genes. In the prognostic analysis, significant differences were found between the upper and lower halves according to the expression levels of several hub genes, including IRF1 (*p* = 0.018), PSMB8 (*p* = 0.038), and RSAD2 (*p* = 0.008).

### 2.4. Construction of the Risk Signature

**Figure 7:**
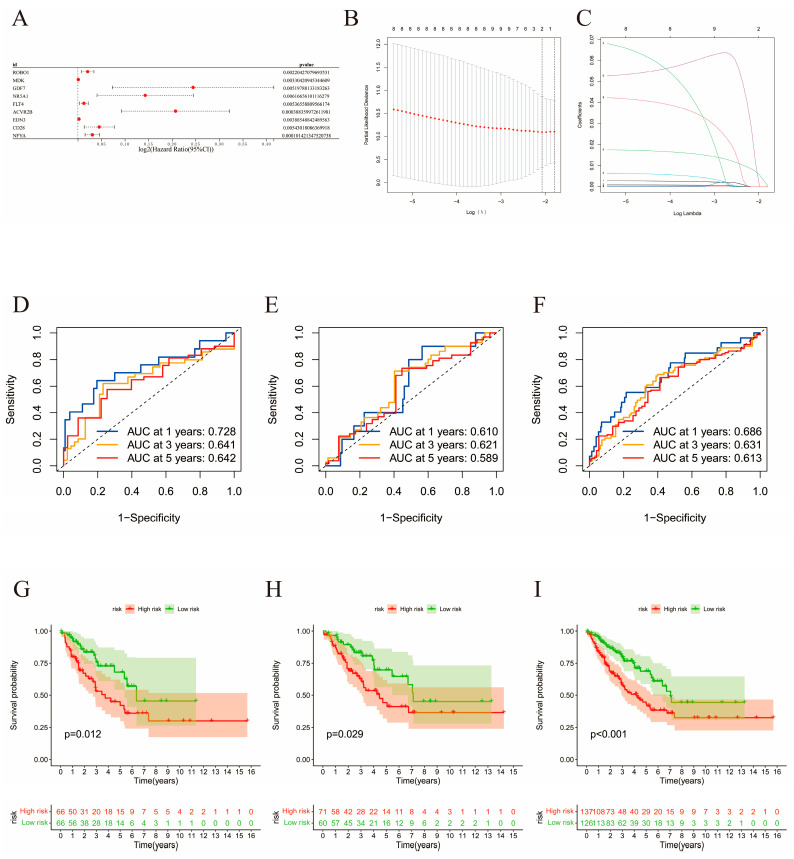
Construction of total risk signatures. (**A**) Forest plot of univariate analyses for nine significant immune-related DEGs of total immune infiltration with overall survival. (**B**,**C**) LASSO analysis to screen for the most significant candidate genes. (**D**–**F**) ROC curves for prognostic values of risk signature in training, validation, and the initial merged cohort, respectively. (**G**–**I**) K-M curves of survival analysis for training, validation, and the initial merged cohort, respectively.

**Figure 8:**
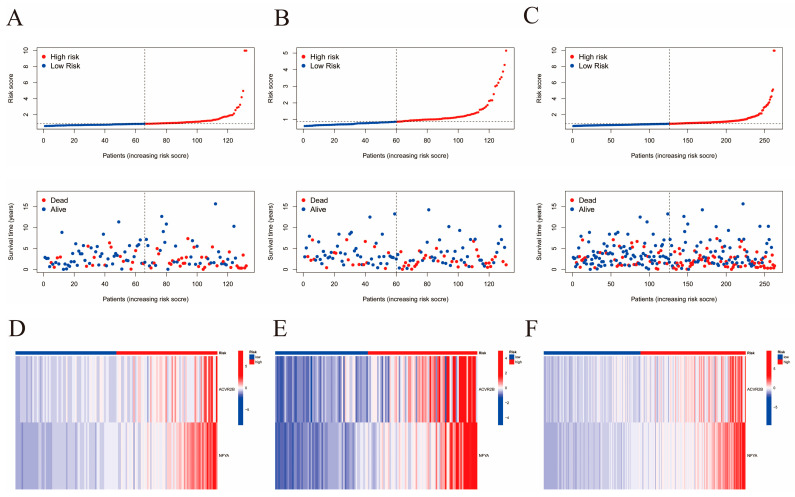
Analysis of correlation between high- and low-risk scores with survival and candidate gene expression. (**A**–**C**) Correlation between risk score and survival rate, based on training, validation, and the initial merged cohort, respectively. (**D**–**F**) Expression level of the two selected genes in the high- and low-risk groups, respectively, based on training, validation, and the initial merged cohort.

**Figure 9:**
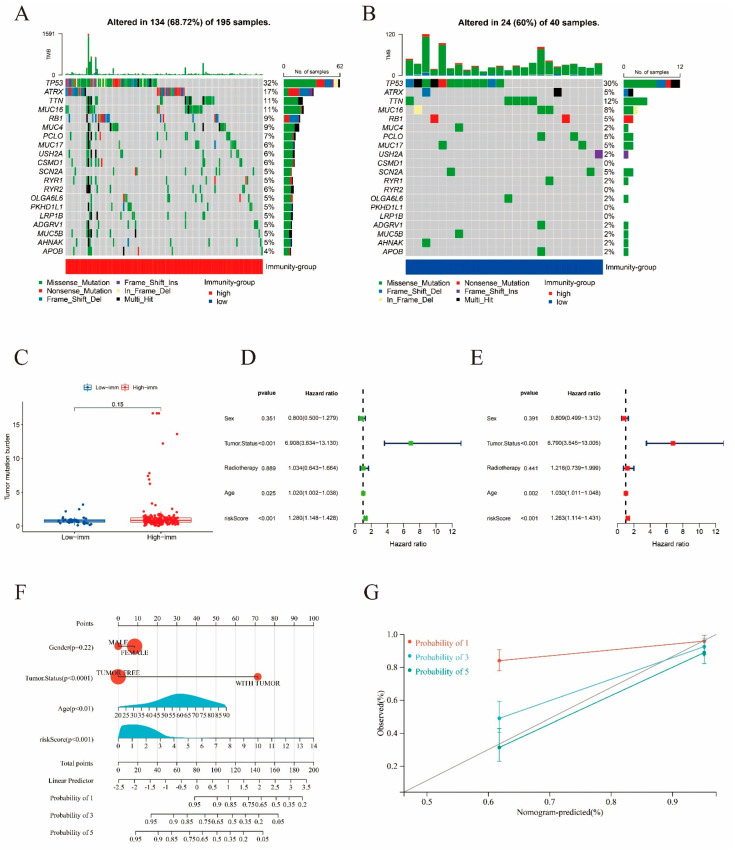
Mutation analysis and validation of the risk signature. (**A**,**B**) Mutation rate analysis performed on immunity−high and −low groups, with colors red and blue representing immunity−high and −low groups, respectively. (**C**) Tumor mutation burden of immunity−high and −low groups. (**D**,**E**) Forest plots of the univariate and multivariate Cox analysis for the risk score and clinicopathological parameters (sex, tumor status, radiotherapy, age) with overall survival. (**F**) Nomogram including clinical characteristics including gender, tumor status, age, and risk score. The “total score” axis corresponds to the 1−, 3−, and 5−year overall survival probabilities on the lower axis, representing the prognostic survival of sarcoma patients with distinct total scores for 1, 3, and 5 years. (**G**) Calibration curves for nomogram.

The 263 sarcoma samples were randomly and evenly distributed between the training and validation cohorts. Using univariate Cox analysis (*p* < 0.0062), 9 genes out of 590 immune-related DEGs were selected as favorable factors with prognostic potential for further analysis (Table S9). Using least absolute shrinkage and selection operator (LASSO) multivariate modeling, two candidate genes (*ACVR2B* and *NFYA*) were obtained to establish the risk signature (Table S10, Figure 7A–C). According to the expression levels and coefficient values of the candidates, a risk score model was developed using the following formula: risk score = (0.1049 × expression of *ACVR2B*) + (0.0188 × expression of *NFYA*). The formula was applied to both cohorts to obtain the risk score for each sample. Receiver operating characteristic (ROC) analyses were then also performed for both cohorts, as well as the original merged cohort (Figure 7D–F). An area under curve (AUC) value of a ROC curve larger than 0.5 is considered statistically significant. ROC analyses for training, validation, and the merged cohort all presented AUC values greater than 0.5, while AUC at 1 year was the largest, with areas of 0.728, 0.610, and 0.686, respectively. These results suggest that the signature exhibited high sensitivity at 1, 3, and 5 years for the training, validation, and both cohorts, respectively. Survival curves were generated for the same three cohorts to evaluate the correlation between the risk score and survival (Figure 7G–I). A significant survival advantage of the low-risk group was demonstrated for all the three cohorts, suggesting a strong association between the low-risk score and high survival possibility. Therefore, this signature was considered prognostically valuable. In further analyses, it was assumed that the overall survival decreased with increasing risk score. Distribution plots of the risk scores were then constructed to verify the above results (Figure 8A–C). To assist in the comprehension of the influence of the two hub genes on oncogenesis, the distributions of the two genes in the training, validation, and original merged groups were analyzed and are illustrated as heat maps (Figure 8D–F). High expression levels of both *ACVR2B* and *NFYA* were in positive correlation with higher risk scores, suggesting that these two genes may act as risk factors for sarcoma development and clinical deterioration. To further understand the genetic features and differences between the immunity-high and immunity-low groups, mutations in the two groups were analyzed separately (Figure 9A,B). Among the 195 samples in the immunity-high group, 134 (68.72%) had mutations in various genes, of which the most frequently mutated gene was *TP53*. In the immunity-low group, 24 out of 40 patients (60%) exhibited gene alterations. The mutation rate in the high-score group was slightly higher than that in the lower half. However, the difference was not statistically significant (*p* = 0.15, Figure 9C).

### 2.5. Visualization and Validation of a Nomogram

To analyze the extent to which clinicopathological parameters contributed to the outcome, and to verify the prognostic capability of the model, univariate and multivariate Cox regression analyses were applied (Figure 9D,E). The risk score constructed in this study exhibited significant value in prognostic prediction within an appropriate amount of time (*p* < 0.001), confirming the possibility of utilizing this risk signature as a prognostic tool. In addition, other clinical factors, including tumor status (*p* < 0.0001) and age (*p* < 0.01), also presented significant influence on the prognosis of patients with sarcoma. With the foundation of the Cox regression analysis, a nomogram comprised of the risk score and other clinicopathological parameters was generated and corrected for the prognostic prediction of survival of patients with sarcoma at 1, 3, and 5 years (Figure 9F,G). The visualization of the prognostic model through a nomogram instead of an equation presents a clearer version of the model, making the evaluation of the prognosis of patients with sarcoma more convenient for clinical usage. To examine the accuracy of the nomogram, calibration curves were generated and presented in Figure 9G. The calibration curves indicate the high accuracy of the nomogram, especially in predicting the 5-year overall survival.

## 3. Discussion

According to recent studies, immune response activation significantly affects the oncogenesis and progression of cancers [18]. To further investigate the underlying mechanisms, DEGs with different immune-related features were screened and analyzed. In this study, most of the immune-related DEGs were downregulated, and showed statistically significant abundance in numerous immune-related biological pathways. Leukocytes play an essential role in immune defense and are considered to be instructors of the immune system. The activation of neutrophils in the TME is regarded as a sign of tumor regression due to the tumor being the immune target [19,20]. Another study discovered that the absolute amount of lymphocyte, neutrophil-lymphocyte ratio, and lymphocyte-monocyte ratio showed potential in identifying patients predisposed to poor outcomes [21]. As the enrichment and activation of lymphocytes and leukocytes were found to be statistically significant in this study, it is possible that the immune characteristics of the sarcoma TME are of prognostic value.

According to the above results, the ESTIMATE score and sarcoma tumor purity exhibited a significant association with the prognosis of patients with sarcomas. Higher immune scores suggested higher immune cell infiltration levels, which resulted in lower tumor purity. The survival rate showed significant correlation with ESTIMATE scores, positively, and tumor purity score, negatively. Subsequently, significantly different tumor purity between the two groups with different levels of immunity were observed. According to the results of GO, KEGG, and GSEA for the two immune subtypes, a considerable number of DEGs showed enrichment in immune-related pathways. Among all of the GO terms of BP, T-cell activation was found to be associated with the highest number of DEGs. Previous studies have suggested that T-cell infiltration and activation exert an important impact on the outcome of various sarcomas. In 2021, Kosei et al. discovered that the interaction between T cells and sarcomas appeared to have an important influence on tumor progression [22]. Adoptive T-cell therapy holds great promise for sarcoma treatment, where activated autologous T cells induce endogenous lymphocyte infiltration, resulting in sustained cancer remission or clearance [22,23]. In our study, the extent of immune cell infiltration of the two groups of sarcomas with different high or low levels of immune activation were investigated, indicating that the group with a high level of immune activation had infiltration levels of various immune cell types that were significantly higher than the group with lower immune-activation, which correlated with the survival advantage seen in the immunity-high group. Ten hub genes were identified in the above analysis, and the association between various immune cell types was examined. All ten hub genes were clearly related to T cells. 

A risk signature was then established based on two immune-related hub genes, *ACVR2B* and *NFYA*, where a low-risk score was found to be associated with a favorable survival and better prognosis. The risk score increased with higher *ACVR2B* and *NFYA* expression levels. Activin A receptor type II B (ACVR2B) is commonly known to be one of the myostatin receptors, and is involved in the negative autocrine/paracrine signaling pathway in skeletal muscle development through the activation of the SMAD 2/3-dependent signal transduction and MAPK pathways, as well as the inhibition of the Akt pathway [24,25]. Regarding oncogenesis, *ACVR2B* is generally believed to be unrelated to the development of tumors, but contributes significantly to cachectic effects that usually accompany the deterioration of disease and the utilization of chemotherapy [26,27,28]. The association between the overexpression of *ACVR2B* and the unfavorable survival revealed by this study is in agreement with previous discoveries. Investigations have been performed on the clinical potentials of the ACVR2B-signaling inhibitors in relieving multi-organ co-morbidities and cachexia-accompanying cancers, side effects of chemotherapy, and muscle damage caused by other diseases, such as ischemic heart failure. In 2010, Margaret E Benny Klimek et al. first identified the potential of ACVR2B-Fc, an artificial fusion protein that has the extracellular domain of the ACVR2B fused to human Fc, acting as an antagonist of the ACVR2B signaling, in attenuating cachemia in both colon-26 and Lewis lung carcinoma, with no influence on the progression of tumors [29]. Since then, the same potential of ACVR2B antagonists, including ACVR2B-Fc and other inhibitors, such as microRNA-194, has also been discovered in different cancers, including pancreatic cancer and clear cell kidney carcinoma [30,31]. Therefore, the association between overexpressed *ACVR2B* and poor prognosis of patients with sarcoma suggests a possibility of utilizing ACVR2B antagonists in reliving the cachemic effect of sarcoma and the therapy-induced loss of muscle mass, which has not yet been investigated.

Nuclear transcription factor Y alpha (NFYA), a CCAAT-binding transcription factor, was found to be responsible for many, if not most, cell cycle regulators, acting as a positive factor for cell proliferation. In 1994 and 1996, Chang et al. and Good et al., respectively, discovered that the NFYA subunit and CCAAT-binding were abundant in young cells, but reduced in older cells [32,33]. The overexpression of this gene is suggested to be of significant importance in various cancers, including breast, ovarian, lung, and gastric cancers, according to recent literature [34,35,36,37,38,39,40]. In 2011, NFYA was found to be involved in the high activity of aldehyde dehydrogenase, a characteristic enzyme of uterine endometrial adenocarcinoma, which is associated with a poor prognosis [35]. In a study in 2017 performed by Cicchillitti et al., the expression level of *NFYA* was identified as a potential biomarker to predict the risk of recurrence of endometrial cancer [36]. In lung cancer, *NFYA* and other CCAAT-related transcription factors were found to be overexpressed, according to the results of Bezzecchi et al. [37]. Similar results for the upregulation of *NFYA* were reported in breast cancer by Dolfini et al. [39]. Based on a study performed by Bie et al. on gastric cancer, the oncogenic effect of *NFYA* resulted from an increase in the transcription of cyclin E and other cell cycle regulatory genes [38]. Therefore, the results obtained in this study are theoretically and practically supported, suggesting that NFYA could also be a potential target for sarcoma therapy. In 2022, Fengyan Han et al. discovered that through inhibiting the nucleocytoplasmic transport of NFYA, the metastasis of colorectal cancer can be suppressed by using delanzomib, an antagonist of the NFYA-related signaling pathway, which further demonstrated the potential of NFYA-targeted chemotherapy for cancer [41]. However, no research has been performed on the potential of NFYA antagonists in sarcoma therapy.

According to the results of GO, KEGG, and GSEA for the two immune subtypes, a considerable number of DEGs were found to be enriched in immune-related pathways. Because the prognostic model designed in our study is highly immune-related, its relevance for immunotherapy for sarcomas should be further investigated. To date, immunotherapies for sarcomas have generally been categorized into two forms, immune checkpoint inhibitors (ICIs) and adoptive cell therapy, both of which involve different levels of clinical trials.

Star ICIs are inhibitors of PD-1/PD-L1, such as pembrolizumab and nivolumab, and anti-CTLA-4 agents, such as ipilimumab. In 2017, a phase-II nonrandomized multi-cohort study of pembrolizumab based on 42 types of sarcomas was performed by Tawbi et al., the results of which demonstrated that 18% of patients experienced an objective or complete response at the median follow-up time [42]. An update to this study was presented online in 2019, demonstrating that a final overall response rate of 23% in patients with undifferentiated pleomorphic sarcoma can be achieved through long-term usage of the PD-1/PD-L1 inhibitor pembrolizumab. Considering CTLA-4 inhibitors, monotherapy with ipilimumab did not show a response rate as high as that of inhibitors of PD-1/PD-L1. There has only been one study on the efficacy of monotherapy with ipilimumab, which was terminated prematurely because no objective response was shown in any patient [43]. On the basis of the above results, it can be seen that the two-star ICIs not only have different mechanisms of action, but also different response rates, which encourages further studies on ICI-related immunotherapy for sarcomas. In contrast to the above agents, which have been extensively studied, to the best of our knowledge, no investigations utilizing adoptive T-cell therapy on sarcomas have been reported. The use of activated immune cells in sarcoma-targeted therapy has been limited to case reports. Nevertheless, T-cell infiltration and activation have been suggested to exert an important impact on the outcomes of various sarcomas, suggesting that adoptive T-cell therapy might hold the key to future immunotherapies for sarcomas.

In our study, among all of the GO terms of BP, T-cell activation was found to be associated with the highest number of DEGs. Previous studies have suggested that T-cell infiltration and activation play important roles in determining the outcomes of various sarcomas. Adoptive T-cell therapy holds great promise for sarcoma treatment, in which activated autologous T cells induce endogenous lymphocyte infiltration, resulting in sustained cancer remission or clearance [22,23]. In our study, the infiltration levels of various immune cell types in the immunity-high group significantly exceeded that of the other group with a lower level of immune activation, which correlated with the survival advantage shown in the immunity-high group. Ten hub genes were identified in the above analysis, and the association between various immune cell types was examined. All 10 hub genes were clearly related to T cells. The accuracy and efficiency of the signature was examined and verified using a nomogram (*p* < 0.001). Across clinical descriptors, including sex, tumor status, radiotherapy, and age, no significant influence was observed (*p* < 0.05). This result further confirms the importance and urgency of establishing a sensitive and accurate risk signature for sarcomas. In Cox analyses, the risk score was calculated as statistically significant with *p* < 0.001, verifying the reliability of the risk signature.

Currently, an increasing number of studies are being conducted to discover better signatures for sarcomas [44,45]. However, risk models for the prognosis of sarcoma patients are insufficient. In 2020, Gu et al. established a risk score model based on the expression of five genes (*TSPAN7*, *MYBL2*, *GCSH*, *FBN2*, and *DDX39B*) for the prognostic prediction of soft tissue sarcoma [46]. In the same year, another model based on eight genes (*KCNJ15*, *SLC24A4*, *ASPA*, *REM1*, *SCARA5*, *LANCL3*, *CPA6*, and *TRH*) for osteosarcoma by Wu et al. was developed. [47]. Although both risk models possessed relatively good features, they did not evaluate the influence of immunity on sarcomas. As demonstrated by the results of the current study, the influence that immune infiltration has on the development of sarcomas is non-negligible. The risk signature constructed in our work is based on immune-related DEGs between immunity-high and immunity-low groups. Our model possesses acceptable accuracy and efficiency, providing a novel and valuable tool for prognostic prediction and inspiration for future research.

## 4. Materials and Methods

### 4.1. Acquisition of Data

Both the clinical and genetic data included for analyses were retrieved from the online database The Cancer Genome Atlas (TCGA) Sarcoma cohort [48]. The transcription data were investigated in fragment per kilobase of transcript per million mapped reads (FPKM) values, which were later transformed into TPM for downstream analyses.

Among the originally retrieved data, samples with no information on gene expression, clinical parameters, or survival status were excluded from further study, leaving us with a total of 263 sarcoma samples and 2 normal samples. Furthermore, we downloaded the somatic mutation data and the copy number variation (CNV) profile of the 263 patients from the same database for further analyses. The immune-associated gene set was obtained from the TIMER database with 1811 immune-related genes for subsequent analysis (Table S1).

### 4.2. Immune Evaluation, Clustering, and Comparison of Immune Characteristics

The presence of immune cells and stromal cells and the estimated tumor purity of the samples were evaluated based on the immune score, stromal score, and ESTIMATE score, and calculated using the Estimation of STromal and Immune cells in MAlignant Tumor tissues using Expression data (ESTIMATE) algorithm [17,49]. In accordance with the mean values of the immune, stromal, and ESTIMATE scores acquired above, the sarcoma samples were divided into two groups according to the median scores, which were then named the high-score group and low-score group, respectively. To explore the correlation between the three parameters achieved from the above analysis and overall survival (OS), survival analyses were performed.

Subsequently, the ssGSEA algorithm was utilized to assess the immune signatures of 30 immune cell types, in order to specify the description of abundance of immune signatures and immune infiltration degree in each sample (Table S2) [50]. Differentiations in multiple characteristics between the immune phenotypes were thoroughly studied. Clinical features, including age, radiotherapy, tumor status, sex, race, event, OS, and other parameters mentioned from a previous analysis of different phenotypes were compared and visualized using a heatmap. In addition, a box plot illustrating the abundance of infiltrating immune cells in distinct immune phenotypes was generated to assist in the comprehension of immune characteristics of different immune phenotypes. Then, the DEGs between distinct immune groups were retrieved using the classical Bayesian approach in the limma package in R software version 4.0.3. With the standard set for screening, *p* < 0.05 and |logFC| > 0.585, genes that fit the description were considered as DEGs between the two immune subtypes. A volcano plot was then constructed to visualize the expression levels of DEGs. To further specify the description of the DEGs on the enrichment of pathways and functional annotation, GO functional annotation and KEGG pathway analysis were performed using the cluster Profiler package [51]. Terms with *p* < 0.05 were selected to present the enrichment results. Gene Set Enrichment Analysis (GSEA) was employed to analyze the differences in pathways between the immunity-high and -low groups [52]. The interactions among DEG-encoded proteins were retrieved from the STRING database and quantified in the form of PPI score.

### 4.3. Immune-Related DEGs Assessment

The identical genes in DEGs and immune-related genes were screened using Venn analysis in limma package VennDiagram in R version 4.0.3. The duplicated genes were named immune-related DEGs. Then, GO functional annotation and KEGG pathways enrichment analysis were conducted for the immune-related DEGs. A PPI network was constructed based on the PPI score of the immune-related DEGs obtained from the STRING database using Cytoscape to reveal the interaction between the immune-related DEGs. Finally, a GSEA analysis were performed for these overlapped genes using the approaches mentioned above. Interactions between these genes was comprehensively analyzed. The hub genes were ranked by the MCC method, with the top 10 genes selected. Subsequently, the immune infiltration and overall survival of high- and low-score groups of the ten hub genes were investigated.

### 4.4. Construction of the Risk Signature

The samples were evenly divided into groups for training (*n* = 132) and validation (*n* = 131) of the model. The division of the samples was strictly random, with no difference in any clinical manifestation of statistical significance between the two groups. Then, the expression levels of the hub genes were correlated with the OS to make further analysis more convenient. A univariate Cox regression analysis was used to screen for survival-associated DEGs among those already found related to immunity. The ones with *p* < 0.0062 were selected as genes with significant correlation. The immune-related DEGs with significant survival association were then subjected to penalized multivariate Cox proportional hazards survival modeling using an algorithm for variable screening based on Least Absolute Shrinkage and Selection Operator (LASSO) estimation to screen for ideal candidate genes [53]. According to the expression (*Expi*) and estimated regression coefficients (*coefi*) of the candidates obtained from the LASSO regression analysis, a risk signature was established using the formula below [54,55]:

(1) $$Risk\;score=\overset{n}{\underset{i=1}{\sum }}coefi\times Expi$$

where *n* is the number of genes identified for the risk signature, *coefi* is the coefficient for the gene, and *Expi* stands for the expression value of the gene. Consequently, a risk signature was established, with the risk score for each sample calculated according to the above formula. Based on the median risk scores, samples were separated into two groups, between which the upper half and lower half were named high- and low-risk groups, respectively. Then, the prognostic performance of the signature was predicted on the basis of the performance of the training, validation, and all groups, which was presented using a time-dependent ROC curve analysis. The high- and low-risk groups were then tested for differences in overall survival for validation, using the same resorts mentioned above.

### 4.5. Tumor Somatic Mutation Analysis

The total amount of unique genes without synonymous somatic mutations per Mb in each sample was examined for tumor mutational burden (TMB). Frame-shift deletion or insertion, nonsense mutation, and splice-site mutation were included in the category of truncating mutation. In-frame deletion or insertion, missense, and nonstop mutation, on the other hand, were included in the non-truncating mutation category.

### 4.6. Prognostic Evaluation of the Signature

To evaluate the risk score and clinical risk factors, univariate and multivariate Cox regression analyses were conducted using the R packages. Subsequently, a nomogram based on the Cox regression analysis was generated for validation of the risk signature and to present the relationships between the variables and the 1-, 3-, 5-year overall survival of patients with sarcoma. Then, to assess the capability of the signature, calibration curves for the nomogram were generated, with the *x*-axis representing the nomogram-predicted probability and the *y*-axis representing the observed probability.

## 5. Conclusions

From the above description of this work, it can be concluded that a high ESTIMATE score and low tumor purity are related to a better prognosis in sarcomas. Several analyses were performed to investigate the association between immune activation, tumor purity, and prognosis. The samples were first categorized into two distinct groups according to their immune scores using ssGSEA. It was then revealed that the group with higher immune activation and TME immune cell infiltration exhibited a significant survival advantage over the other group. A risk signature was then constructed based on the immune-related DEGs obtained from the two immune subtypes using univariate Cox and LASSO analyses. The risk signature was found to have significant prognostic value and is visualized in the form of nomogram. The signature was proven to be accurate and efficient. Therefore, it is suitable for further optimization and eventual clinical use. 

## Data Availability

Data is contained within the article or supplementary material. The data presented in this study are available in Supplement Figure S1, and Supplement Tables S1–S10.

## Supplementary Materials

The following supporting information can be downloaded at: https://www.mdpi.com/article/10.3390/ijms24032961/s1.
