# Supplementary material for: A New Signature of Sarcoma Based on the Tumor Microenvironment Benefits Prognostic Prediction
Source: Int J Mol Sci. 2023 Feb 3;24(3):2961. doi: 10.3390/ijms24032961 (PMC9918054; doi:10.3390/ijms24032961)
Supplement: Supplementary file 1 [file ijms-24-02961-s001.zip › ijms-2056709-supplementary figure.pdf]

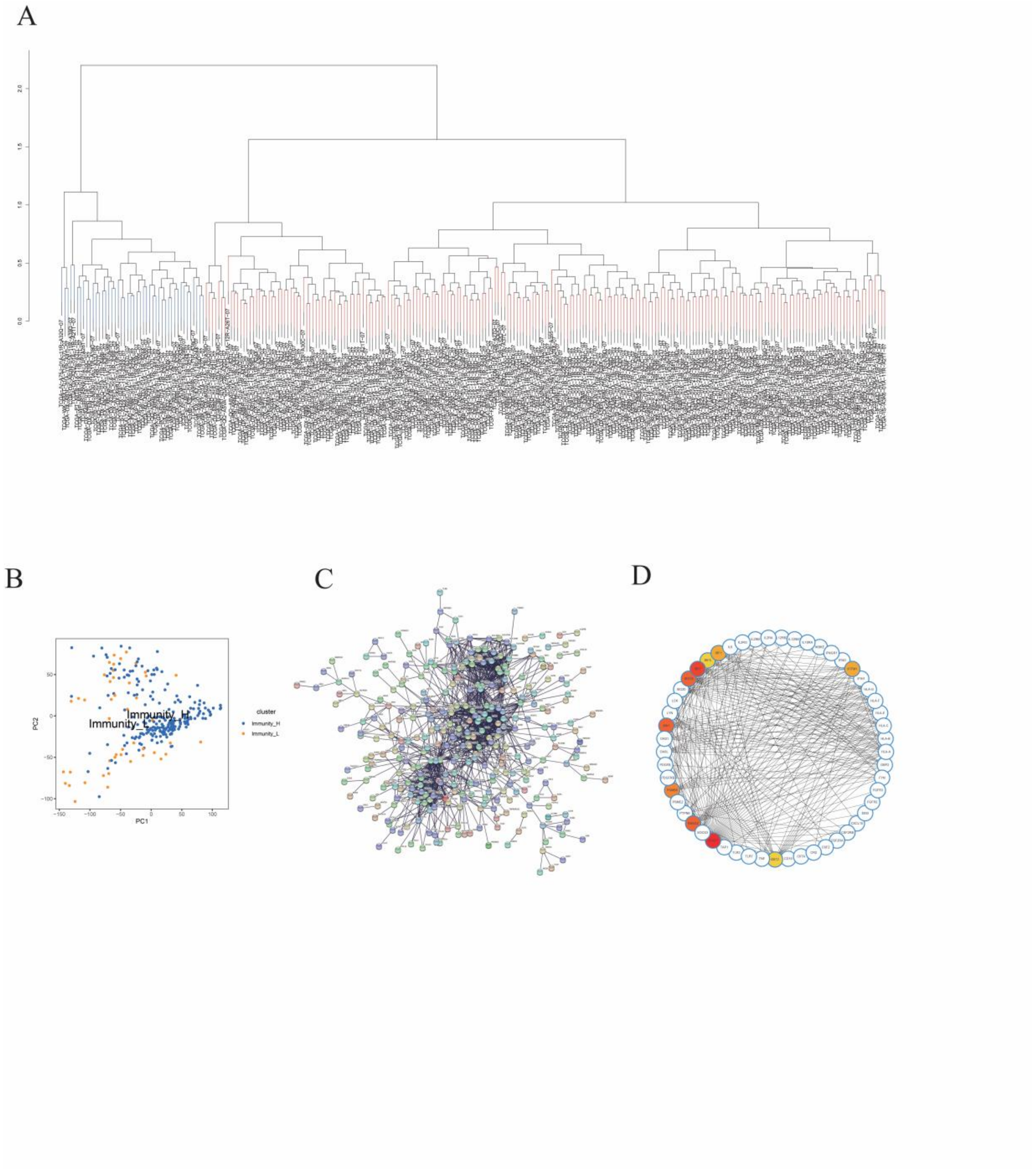


**Figure S1.** (A) hclust graph demonstrating the clustering of sarcoma samples according to immune activation. (B) Result of PCA for immunity-high and -low groups. (C) The PPI network for immune-related DEGs, with the minimum required interaction score as highest confidence (0.900). The line thickness indicates the strength of data support. (D) Interaction between hub genes from immune-related DEGs.
